# Desfechos Hospitalares do Infarto do Miocárdio com Supradesnivelamento do Segmento ST em Pacientes Positivos para COVID-19 que Passaram por Intervenção Percutânea Primária

**DOI:** 10.36660/abc.20230258

**Published:** 2024-01-24

**Authors:** Nart Zafer Baytuğan, Hasan Çağlayan Kandemir, Tahir Bezgin

**Affiliations:** 1 Gebze Fatih State Hospital Gebze Turquia Gebze Fatih State Hospital – Cardiology, Gebze – Turquia; 2 Kocaeli Devlet Hastanesi Kocaeli Turquia Kocaeli Devlet Hastanesi – Cardiology, Kocaeli – Turquia

**Keywords:** COVID-19, Infarto do miocárdio, Mortalidade, Choque cardiogênico

## Abstract

**Fundamento:**

A infecção concomitante por coronavírus 2019 (COVID-19) e o infarto do miocárdio com supradesnivelamento do segmento ST (IAMCSST) estão associados ao aumento de desfechos adversos hospitalares.

**Objetivos:**

O estudo teve como objetivo avaliar as diferenças angiográficas, de procedimentos, laboratoriais e prognósticas em pacientes positivos e negativos para COVID-19 com IAMCSST submetidos à intervenção coronária percutânea primária (ICP).

**Métodos:**

Realizamos um estudo observacional retrospectivo e unicêntrico entre novembro de 2020 e agosto de 2022 em um hospital de nível terciário. De acordo com o seu estado, os pacientes foram divididos em dois grupos (positivo ou negativo para COVID-19). Todos os pacientes foram internados por IAMCSST confirmado e foram tratados com ICP primária. Os desfechos hospitalares e angiográficos foram comparados entre os dois grupos. P-valores bilaterais <0,05 foram aceitos como estatisticamente significativos.

**Resultados:**

Dos 494 pacientes com IAMCSST inscritos nesse estudo, 42 foram identificados como positivos para COVID-19 (8,5%) e 452, como negativos. Os pacientes que testaram positivos para COVID-19 tiveram um tempo isquêmico total maior do que os pacientes que testaram negativos para COVID-19 (p = 0,006). Além disso, esses pacientes apresetaram um aumento na trombose de stent (7,1% vs. 1,7%, p = 0,002), no tempo de internação (4 dias vs. 3 dias, p = 0,018), no choque cardiogênico (14,2% vs. 5,5%, p = 0,023) e na mortalidade hospitalar total e cardíaca (p <0,001 e p = 0,032, respectivamente).

**Conclusões:**

Pacientes com IAMCSST com infecções concomitantes por COVID-19 foram associados ao aumento de eventos cardíacos adversos maiores. Mais estudos são necessários para compreender os mecanismos exatos dos desfechos adversos nesses pacientes.

**Figura Central:**
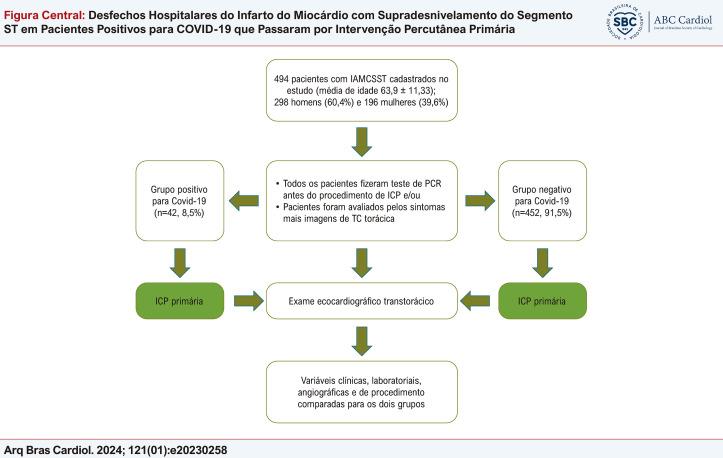
: Desfechos Hospitalares do Infarto do Miocárdio com Supradesnivelamento do Segmento ST em Pacientes Positivos para COVID-19 que Passaram por Intervenção Percutânea Primária

## Introdução

A doença do coronavírus 2019 (COVID-19), causada pelo coronavírus 2 (SARS-CoV-2), é uma pandemia desde março de 2020 e tornou-se uma crise de saúde global em pouco tempo. ^1 , 2^ Embora a COVID-19 afete principalmente o sistema respiratório, pode levar ao envolvimento de múltiplos órgãos, infecção sistêmica, disfunção endotelial vascular, infarto do miocárdio e morte. ^3^ Infecções virais sistêmicas e hipóxia podem desencadear ativação plaquetária, ruptura de placas e síndromes coronárias agudas com distúrbio do sistema endotelial vascular. ^4 , 5^

A COVID-19 afeta os sistemas arterial e venoso e leva a um aumento da atividade trombótica. A trombose é uma complicação grave que frequentemente se manifesta como embolia pulmonar, infarto cerebral e tromboembolismo venoso. Síndrome coronária aguda, isquemia mesentérica e cerebrovascular e trombose da artéria renal são menos comuns. O infarto do miocárdio com supradesnivelamento do segmento ST (IAMCSST) geralmente ocorre como resultado de oclusão trombótica completa da artéria coronária e requer diagnóstico rápido e estratégia de reperfusão. Não há dados suficientes sobre o efeito da coinfecção por COVID-19 nos desfechos clínicos de pacientes com IAMCSST.

Planejamos um estudo retrospectivo, unicêntrico, para avaliar as variáveis clínicas, angiográficas, laboratoriais e de procedimento em pacientes positivos para COVID-19 com IAMCSST em comparação com pacientes com IAMCSST e negativos para COVID-19.

## Métodos

### População de pacientes

Realizamos um estudo observacional, unicêntrico e retrospectivo entre novembro de 2020 e agosto de 2022. Um total de 494 pacientes consecutivos com IAMCSST admitidos em nosso laboratório de cateterismo foram incluídos no estudo. O IAMCSST foi definido com base no supradesnivelamento do segmento ST em duas ou mais derivações contíguas ≥ 0,2 mV ou novo bloqueio de ramo esquerdo associado a um novo aparecimento de dor torácica. ^6^ O diagnóstico angiográfico de doença coronariana oclusiva foi realizado em todos os pacientes e nenhum foi tratado com fibrinólise. Os dados dos pacientes foram obtidos de registros do banco de dados do hospital. Exames laboratoriais (hemograma completo, parâmetros inflamatórios e parâmetros bioquímicos) foram realizados em todos os pacientes na admissão. Pacientes sem IAMCSST, sem dados suficientes e com parada cardíaca foram excluídos do estudo. Além disso, pacientes que não foram submetidos a intervenção coronária percutânea (ICP) (lesões não obstrutivas das artérias coronárias, vasoespasmos ou cirurgia de revascularização miocárdica de emergência dirigida) não foram avaliados. Este estudo foi realizado de acordo com os princípios da Declaração de Helsinki, e o comitê de ética local aprovou o protocolo do estudo. A ilustração central apresenta o protocolo do estudo.

### Coleta de dados clínicos

Amostras de swab nasal foram coletadas de todos os pacientes na sala de emergência ou laboratório de cateterismo antes da ICP. A infecção por COVID-19 foi confirmada por ensaios de reação em cadeia da polimerase via transcriptase reversa em tempo real (RT-PCR) e/ou pela avaliação dos sintomas com tomografia computadorizada (TC) torácica. Eles foram categorizados como positivos ou negativos para COVID-19. Tratamentos adicionais (antibióticos, antivirais, etc.) foram iniciados utilizando as abordagens atuais nos pacientes positivos para COVID-19.

A fração de ejeção do ventrículo esquerdo (FEVE) foi medida usando uma imagem 2D do volume diastólico final e do volume sistólico final pelo método de Simpson modificado. As alterações valvares foram avaliadas como regurgitação moderada ou grave ou estenose das valvas mitral ou aórtica.

Foram registrados a condição clínica dos pacientes, história adicional de doença, tabagismo, tempo de internação, trombose de stent (TS), hemorragia, choque cardiogênico e taxa de mortalidade. Além disso, o uso de inibidores da glicoproteína IIb-IIIa, cateteres de aspiração e bombas de balão intra-aórtico foi analisado retrospectivamente. O tempo de início dos sintomas e o tempo porta-balão foram registrados para todos os pacientes. As imagens angiográficas coronarianas foram analisadas como lesão responsável e tipo de lesão, no-reflow pós-ICP, fenômeno de fluxo lento e lesão residual por dois cardiologistas especialistas diferentes, cegos em relação aos dados do paciente.

### Procedimentos angiográficos

Os procedimentos foram realizados de acordo com as diretrizes atuais, e a escolha da artéria radial ou femoral, estratégia de colocação do stent, pré-dilatação, pós-dilatação, uso de inibidores da glicoproteína IIb-IIIa e cateter de aspiração ficaram a critério do operador. A terapia antiagregante dupla foi iniciada em todos os pacientes antes do procedimento. As TS aguda e subaguda foram definidas dentro de 24 horas e 1 mês após o implante do stent, respectivamente, de acordo com as definições do Academic Research Consortium. ^7^ Todos os pacientes receberam heparina não fracionada em dose de ataque de 70-100 u/kg com tempo de coagulação ativado >250 segs.

### Definições de desfechos clínicos

Os pacientes foram divididos em dois grupos, positivo ou negativo para COVID-19, comparados de acordo com o tempo de internação, eventos cardíacos adversos maiores (ECAM), hemorragias maiores e menores, choque cardiogênico e taxas de mortalidade hospitalar. Os ECAM foram definidos como infarto do miocárdio, acidente vascular cerebral, insuficiência cardíaca e/ou morte por doença cardiovascular. A designação de hemorragia foi realizada usando as definições do Consórcio de Pesquisa Acadêmica de Sangramento (BARC). ^8^ O choque cardiogênico foi confirmado como sinal de má perfusão de órgãos-alvo, além de pressão arterial sistólica abaixo de 90 mmHg por pelo menos 30 minutos devido à disfunção cardíaca.

### Análise estatística

As variáveis categóricas foram expressas como números e porcentagens e comparadas entre os grupos usando-se o teste qui-quadrado e o teste exato de Fisher. O teste Kolmogorov–Smirnov foi usado para determinar se os dados tinham distribuição normal. Variáveis contínuas com distribuição normal foram expressas como média ± desvio padrão e as com distribuição não normal foram expressas como mediana e faixa interquartil. O teste t de Student não pareado e o teste U de Mann-Whitney foram usados para comparar variáveis contínuas com distribuição normal e não normal, respectivamente. Os parâmetros foram analisados usando análises de regressão logística univariada ou multivariada. O método de entrada foi utilizado na análise univariada e os parâmetros com valores de p <0,1 foram incluídos no modelo de regressão logística multivariada. O modelo multivariado foi ajustado para idade, troponina I cardíaca de alta sensibilidade (cTnI-us), ferritina, hemoglobina, dímero D, COVID-19 (+), tempo isquêmico total (TIT) e tempo porta-balão. Modelos de regressão logística multivariada com variáveis clinicamente relevantes foram realizados para detectar preditores independentes de ECAM. Os métodos retroativos utilizaram análise de regressão logística multivariada e um valor de p <0,05 foi considerado estatisticamente significativo. Para analisar a correlação entre os níveis de dímero D e o fluxo TIMI pós-ICP em pacientes com IAMCSST positivos para COVID-19, foi calculado o coeficiente de correlação de Spearman. Todos os testes tiveram valor p bilateral <0,05 e foram aceitos como estatisticamente significativos. Os dados foram analisados utilizando o SPSS versão 22.0 (SPSS Inc., Chicago, Illinois, EUA).

## Resultados

Foram incluídos no estudo 494 pacientes consecutivos, 298 homens (60,4%) e 196 mulheres (39,6%), com idade mediana de 59 (42-80). Os parâmetros demográficos, clínicos e laboratoriais de linha de base da população do estudo são apresentados na Tabela 1 . O grupo positivo para COVID-19 era mais velho e apresentava prevalência semelhante de tabagismo, hipertensão, doença arterial coronariana, insuficiência cardíaca congestiva, histórico de ICP e fibrilação atrial. Diabetes mellitus (DM) e doença pulmonar obstrutiva crônica (DPOC) foram mais comuns no grupo positivo para COVID-19 ( Tabela 1 ).

**Tabela 1:** – Características demográficas, clínicas e laboratoriais dos pacientes na linha de base

	Negativo para COVID-19 (n=452, 91,5%)	Positivo para COVID-19 (n=42, 8,5%)	p-valor
**Idade**	58 (41-82)	72 (58-83)	<0,001
**Sexo (Feminino), n (%)**	178 (39,3)	18 (42)	0,214
**Tabagismo, n (%)**	189 (41,8)	15 (35,7)	0,515
**Sintomas**			
Dor	383 (84,7)	28 (66,6)	
Dispneia	52 (11,5)	11 (26,1)	
Parada cardíaca	8 (1,7)	2 (4,7)	
Outros	9 (2)	1 (2,3)	
**Histórico médico**			
DM, n (%)	60 (13,2)	18 (42,8)	0,017
HT, n (%)	130 (28,7)	15 (35,7)	0,344
ICC prévia, n (%)	18 (3,9)	3 (7,1)	0,245
DAC prévia, n (%)	38 (8,4)	3 (7,1)	0,431
ICP prévia, n (%)	51 (11,2)	6 (14,2)	0,795
FA, n (%)	24 (5,3)	2 (4,7)	0,712
DPOC, n (%)	68 (15,0)	18 (42,8)	0,021
**Apresentação do IAMCSST**			
Anterior/BRE, n (%)	219 (48,4)	23 (54,7)	
Inferior, n (%)	168 (37,1)	16 (38,0)	
Lateral, n (%)	34 (7,5)	3 (7,1)	
Posterior, n (%)	31 (6,8)	0 (0)	
**Características ecocardiográficas**			
FEVE (%)	45,7 ± 7,1	40,9 ± 8,2	0,009
Doença valvar, n (%)	49 (10,8)	5 (11,9)	0,341
**Classificação Killip**			
Killip I, n (%)	304 (67,2)	18 (42,8)	0,058
Killip II, n (%)	92 (20,3)	10 (23,8)	0,267
Killip III, n (%)	31 (6,8)	7 (16,6)	0,034
Killip IV, n (%)	25 (5,5)	6 (14,2)	0,023
**Valores laboratoriais**			
cTnI-us, ng/mL	1126 (215-32100)	12742 (453-48756)	<0,001
Creatinina, mg/dL	0,8 (0,5-2,2)	0,9 (0,6-2,4)	0,779
Glicemia, mg/dL	105 (79-207)	144 (106-321)	<0,001
AST mg/dL	23 (18-30)	32 (22-47,5)	<0,001
ALT mg/dL	26 (16-29)	28 (18-30)	0,208
Dímero D, ng/mL	0,52 (0,3-1,1)	2,45 (0,8-7,5)	<0,001
Ferritina (ng/ml)	174 (101-316)	421 (134-879)	<0,001
PCR-as (mg/dL)	9,6 (2,3-45,3)	48,9 (25,1-155,9)	<0,001
WBC x10 ^3^ /µL	6810 ± 2617	14820 ± 4321	<0,001
Hemoglobina, g/dL	13,2 ± 1,6	13,4 ± 2,6	0,447
Trombócitos x10 ^3^ /µL	235 ± 81	242 ± 129	0,344
Linfócitos x10 ^3^ /µL	2,1 ± 0,9	0,9 ± 0,7	<0,001

*FA: fibrilação atrial; ALT: alanina aminotransferase; AST: aspartato aminotransferase; DAC: doença arterial coronariana; ICC: insuficiência cardíaca crônica; DPOC: doença pulmonar obstrutiva crônica; DM: diabetes mellitus; BRE: bloqueio de ramo esquerdo; FEVE: fração de ejeção ventricular esquerda; PCR-us: Proteína C. reativa ultrassensível; cTnI-us: Troponina cardíaca I ultrassensível; HT: hipertensão; ICP: intervenção coronária percutânea; WBC: leucócitos.*

### Achados laboratoriais

De acordo com exames laboratoriais na admissão, os pacientes positivos para COVID-19 apresentavam níveis mais elevados de marcadores inflamatórios (cTnI-us, dímero D, PCR-us, ferritina, contagem de leucócitos), glicemia de jejum e níveis de AST. As contagens de ALT, hemoglobina, creatinina e trombócitos foram semelhantes em ambos os grupos ( Tabela 1 ).

### Achados da angiografia coronária

**Figura 1:**
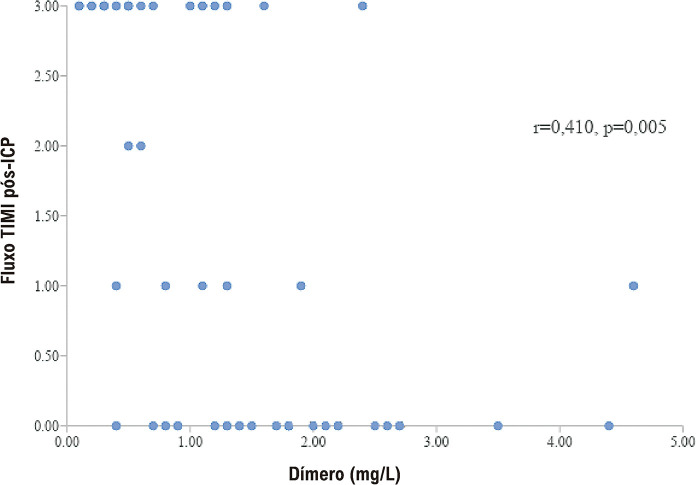
– Correlação entre os níveis de dímero D e o fluxo TIMI pós-ICP em pacientes com IAMCSST positivos para COVID-19.

O diâmetro médio do stent e o comprimento total do stent foram semelhantes em ambos os grupos, e os tamanhos mais curto e mais longo dos stents foram 8 e 56 mm, respectivamente ( Tabela 2 ). A taxa de ICP multiarterial, lesão de bifurcação, pré-dilatação, pós-dilatação, stent sobreposto e proporção de lesão residual foram paralelas nos grupos. Além disso, as taxas de fluxo TIMI 0-1 na linha de base e TIMI 3 pós-ICP foram semelhantes ( Tabela 2 ). O grupo positivo para COVID-19 fez mais uso do inibidor de glicoproteína Iib/IIIa e de dispositivo de aspiração. O fenômeno de no-reflow foi mais alto em pacientes positivos para COVID-19, e o fluxo TIMI 3 pós-ICP e os níveis de dímero D apresentaram correlação negativa ( Figura 1 ). Não houve diferenças entre os grupos em termos de uso de bomba de balão intra-aórtico

**Tabela 2:** – Achados angiográficos e de procedimentos dos pacientes

	Negativo para COVID-19 (n= 452)	Positivo para COVID-19 (n= 42)	p-valor
**Intervenção coronária total, n (%)**	471	44	
**Lesão alvo, n (%)**			
ADAE	216 (47,7)	23 (54,7)	
Cx	97 (21,4)	6 (14,2)	
ACD	123 (27,2)	11 (26,1)	
ACEP	3 (0,6)	0 (0)	
ICP de enxerto	11 (2,4)	0 (0)	
ICP multivascular	19 (4,2)	2 (4,7)	0,680
Lesão de bifurcação	46 (10,1)	3 (7,1)	0,492
**Tipo de lesão, n (%)**			
Tipo A	48 (10,6)	6 (14,2)	
Tipo B	216 (47,7)	24 (57,1)	
Tipo C	188 (41,5)	12 (28,5)	
**Comprimento total do stent (mm)**	26,1 ± 7,0	30,1 ± 6,7	0,319
**Terapia antiagregante**			
Clopidogrel, n (%)	108 (23,8)	19 (45,2)	
Tigacrelor, n (%)	221 (48,8)	18 (42,8)	
Prasugrel, n (%)	123 (27,2)	5 (11,9)	
**Diâmetro médio do stent (mm)**	3,0 ± 0,3	3,0 ± 0,2	0,473
**Stent de sobreposição, n (%)**	32 (6,1)	2 (9,5)	0,815
**Lesão residual, n (%)**	4 (0,8)	0 (0)	0,910
**Fluxo TIMI 0-1 na linha de base, n (%)**	401 (88,7)	40 (95,2)	0,141
**Fluxo TIMI 3 pós-ICP, n (%)**	389 (86,0)	34 (80,9)	0,576
**No-reflow pós-ICP, n (%)**	34 (7,5)	8 (19)	0,043
**Uso de inibidor Gp IIb-IIIa, n (%)**	58 (12,8)	12 (28,5)	0,026
**Uso de trombectomia por aspiração, n (%)**	78 (17,2)	14 (33,3)	0,032
**Uso de BIA, n (%)**	15 (3,3)	4 (9,5)	0,207
**Tempo porta-balão em minutos [FIQ mediano]**	46 (30-72)	48 (36-77)	0,240
**TIT em minutos [FIQ mediano]**	270 (110-670)	390 (180-960)	0,006

*Cx: artéria circumflexa; Gp: glicoproteína; BIA: bomba de balão intra-aórtico; FIQ: faixa interquartil; ADAE: artéria descendente anterior esquerda; ACEP: artéria coronária esquerda principal; ICP: intervenção coronária percutânea; ACD: artéria coronária direita; TIT: tempo isquêmico total.*

### Desfechos hospitalares

**Figura 2:**
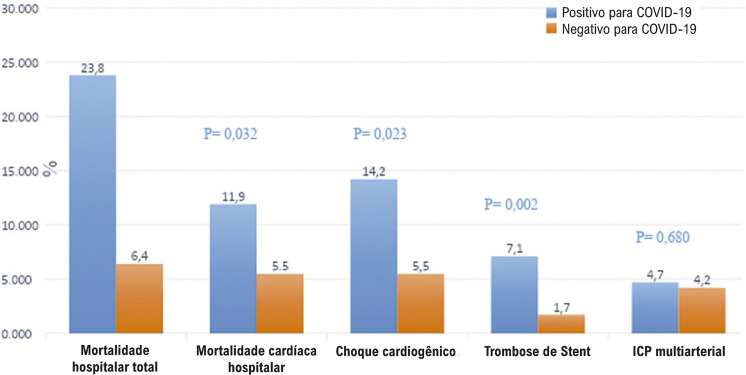
– Desfechos hospitalares com coorte do estudo.

Pacientes com COVID-19 e IAMCSST apresentaram maior mortalidade cardíaca e global intra-hospitalar, TS e choque cardiogênico ( Tabela 3 , Figura 2 ). Na análise multivariada, idade avançada, infecção por COVID-19, dímero D, ferritina, hemoglobina e níveis de cTnI-us foram preditores independentes de ECAM ( Tabela 4 ).

**Tabela 3:** – Desfechos hospitalares dos pacientes

	Negativo para COVID-19 (n= 452)	Positivo para COVID-19 (n= 42)	p-valor
**Choque cardiogênico, n (%)**	25 (5,5)	6 (14,2)	0,023
**Hospitalização total (dia)**	3 (2-6)	4 (3-11)	0,018
**Graus BARC 0-1, n (%)**	14 (3,0)	2 (4,7)	0,372
**Graus BARC 2-4, n (%)**	2 (0,4)	0 (0)	0,571
**Trombose de stent, n (%)**	8 (1,7)	3 (7,1)	0,002
**Mortalidade hospitalar, n (%)**	29 (6,4)	10 (23,8)	<0,001
**Causas de mortalidade**			
Cardíacas, n (%)	25 (5,5)	5 (11,9)	0,032
Sepse, n (%)	-	2 (4,7)	
Falência múltipla de órgãos, n (%)	3 (0,6)	1 (2,3)	
Insuficiência respiratória aguda, n (%)	-	1 (2,3)	
Outras, n (%)	1 (0,2)	1 (2,3)	

**Tabela 4:** – Análises univariadas e multivariadas para previsão dos ECAM

Variáveis	Univariada		p-valor	Multivariada		p-valor
	FC	[IC95%]		FC	[IC95%]	
**Idade**	1,041	1,031-1,052	<0,001	1,023	1,010-1,032	0,003
**Sexo masculino**	0,610	0,382-0,871	0,041			
**Hipertensão**	2,421	1,041-3,080	0,026			
**Diabetes mellitus**	0,901	0,871-1,012	0,070			
**ICC**	1,376	0,954-2,001	0,002			
**cTnI-us**	2,581	1,557-4,280	<0,001	2,466	1,422- 4,263	<0,001
**Creatinina**	0,452	0,181-1,103	0,778			
**PCR-us**	1,532	1,062-2,216	<0,001			
**Ferritina**	1,371	0,952-2,009	<0,001	1,221	0,816-1,837	0,009
**Dímero D**	0,169	0,028-1,222	<0,001	0,244	0,033-1,952	<0,001
**Hemoglobina**	0,900	0,814-1,011	0,007	1,012	0,833-1,205	0,002
**COVID-19 (+)**	3,921	2,051-7,472	<0,001	3,431	1,732-6,825	<0,001
**FEVE**	2,106	1,433-3,092	0,032			
**Doença multivascular**	1,621	0,982-2,688	0,331			
**Uso de BIA**	1,004	1,001-1,102	0,002			
**Uso de inibidor Gp IIb-IIIa**	1,786	1,055-3,012	0,003			
**TIT**	1,344	0,957-1,880	<0,001	1,228	0,811-1,832	<0,001
**Tempo porta-balão**	2,588	1,553-4,287	<0,001	2,466	1,422- 4,260	<0,001

*ICC: insuficiência cardíaca crônica; Gp: glicoproteína; BIA: bomba de balão intra-aórtico; FEVE: fração de ejeção ventricular esquerda; TIT: tempo isquêmico total; PCR-us: proteína C reativa ultrassensível; cTnI-us: troponina cardíaca I ultrassensível.*

Pacientes positivos para COVID-19 tiveram maior tempo de internação hospitalar e apresentaram alta taxa de classe Killip III e IV na admissão hospitalar. A avaliação ecocardiográfica mostrou que os pacientes negativos para COVID-19 apresentaram FEVE mais elevada. Não houve diferença entre os grupos devido a doença valvar ( Tabela 1 ).

**Figura 3:**
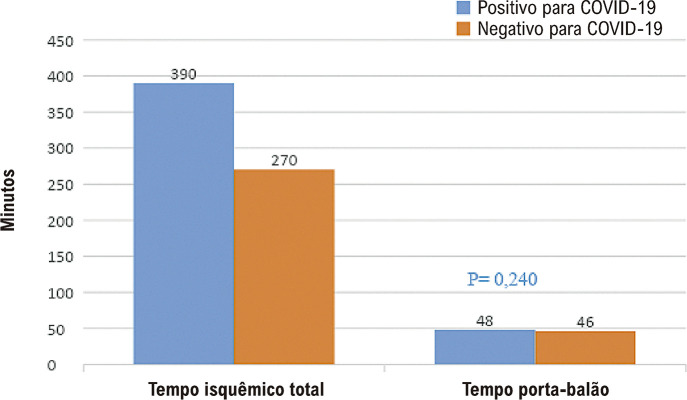
– Tempo isquêmico total e tempo porta-balão nos grupos positivo e negativo para COVID-19.

Os tempos porta-balão foram de 48 minutos e foram semelhantes em ambos os grupos. Entretanto, o TIT foi significativamente mais alto em pacientes positivos para COVID-19 ( Tabela 2 , Figura 3 ). Tanto o tempo porta-balão quanto o TIT foram preditores independentes de ECAM hospitalar ( Tabela 4 ). Não houve diferenças significativas entre os grupos nos graus BARC para hemorragia ( Tabela 3 ).

## Discussão

Planejamos um estudo observacional baseado na experiência de um único centro com alta densidade de pacientes. Esses resultados demonstraram que pacientes com IAMCSST positivos para COVID-19 tiveram uma taxa significativamente aumentada de mortalidade hospitalar cardíaca e total, choque cardiogênico e internação hospitalar. Semelhante ao presente estudo, os achados da literatura atual sugerem que pacientes com COVID-19 apresentando IAMCSST apresentaram taxas mais altas de mortalidade hospitalar e choque cardiogênico. ^9 , 10^

O alto índice de ECAM nesses pacientes pode se dever a várias razões. A alta prevalência de DM e DPOC pode ter contribuído para os maus desfechos nesse grupo. Além disso, o aumento das infecções sistêmicas devido à própria COVID-19 conduziu ao aumento da mortalidade e dos ECAM.

Apesar dos tempos porta-balão semelhantes, houve uma diferença significativa nos níveis de cTnI-us entre os grupos. As possíveis razões para a elevação da cTnI-us em pacientes positivos para COVID-19 incluem TIT mais longo e consequente atraso no diagnóstico correto. A ICP tardia e o aumento do tempo isquêmico podem contribuir para o aumento das enzimas cardíacas. As complicações cardíacas podem ser responsáveis por aproximadamente 40% das mortes em pacientes com COVID-19. ^11^ Aumento da inflamação, hipercoagulação, insuficiência respiratória progressiva, hipóxia, miocardite e efeitos tóxicos diretos do vírus nas células hospedeiras podem levar a danos cardíacos. Além disso, o fenômeno de no-reflow pós-ICP foi significativamente mais comum no grupo positivo para COVID-19, o que pode afetar a perfusão pós-procedimento e levar ao aumento das enzimas cardíacas. Portanto, insuficiência cardíaca e choque cardiogênico podem ser mais comuns nesses pacientes, o que pode explicar os elevados níveis de ECAM, classe Killip e cTnI-us. ^12^ Consideramos que causas não cardíacas em pacientes com COVID-19, como sepse, embolia pulmonar e falência de múltiplos órgãos, podem contribuir para o aumento dos níveis de cTnI-us.

Hipoxemia, vasoconstrição e oxigenação prejudicada são achados comuns na doença COVID-19, portanto, a internação hospitalar é prolongada e há um risco avançado de falência múltipla de órgãos do paciente, infecções bacterianas, sepse e trombose. ^13^ A inflamação sistêmica ativa a cascata pró-trombótica e perturba a função endotelial, aumentando assim o risco de trombose e complicações relacionadas. ^14^ Marcadores inflamatórios elevados estão associados ao aumento das taxas de mortalidade. ^14^ Foi demonstrado que o aumento da resposta inflamatória e as alterações hemodinâmicas aumentam o risco de ruptura da placa e infarto do miocárdio relacionado na infecção pelo vírus influenza. ^15^ Descobrimos que os níveis de PCR-us, ferritina e dímero D foram preditores independentes do desenvolvimento dos ECAM. Além disso, os parâmetros inflamatórios foram significativamente maiores no grupo positivo para COVID-19, semelhantes a estes resultados.

Em nosso estudo, marcadores indicando aumento da atividade trombótica, como trombo multiarterial, TS, fenômeno de no-reflow, uso de inibidores de GP IIb/IIIa e dispositivo de aspiração, foram detectados com mais frequência em pacientes positivos para COVID-19. Uma maior carga de trombos em pacientes com COVID-19 está associada a um risco aumentado de eventos cardíacos adversos e morte. ^16^ Além disso, a embolização distal do trombo pode interromper o fluxo microvascular, levando ao fenômeno de no-reflow e fluxo lento e a um aumento na área infartada. ^17^

Em um estudo realizado por Choudry et al., os parâmetros inflamatórios foram maiores em pacientes com IAMCSST e COVID-19, e os níveis de dímero D estavam correlacionados com o grau do trombo. ^18^ Resultado semelhante foi encontrado em outro estudo, uma correlação positiva entre o grau do trombo e os níveis de dímero D em pacientes com IAMCSST. ^19^ Encontramos um achado paralelo com os níveis de dímero D e uma correlação negativa com o fluxo coronariano pós-ICP. Além disso, foi demonstrado o benefício de mortalidade do uso de anticoagulantes em um grande grupo de pacientes com COVID-19 sem infarto do miocárdio. ^20^

O TIT é um critério importante que afeta a mortalidade em pacientes com IAMCSST. ^21^ O prolongamento desse período reduz o salvamento miocárdico e aumenta a área infartada e subsequente mortalidade em longo prazo. ^21^ As diretrizes atuais recomendam ICP primária com tempo porta-balão de 90 minutos se o paciente se apresentar em um hospital com capacidade para realizar ICP. ^22 , 23^ Onder et al. mostraram que o tempo médio desde o início dos sintomas do IAMCSST até o primeiro contato médico durante a pandemia de COVID-19 foi de 318 minutos, ^24^ e outro estudo de Abdelaziz et al. demonstrou que esse tempo foi em média de 227 minutos. ^25^ Em nosso estudo, descobrimos que esse tempo foi de 390 minutos em pacientes positivos para COVID-19 e foi significativamente mais longo em comparação com pacientes negativos para COVID-19. Descobrimos que o prolongamento do tempo para o primeiro contato médico pode ter afetado o aumento de ECAM observados no grupo positivo para COVID-19 ( Tabela 4 ).

As admissões por IAMCSST em centros médicos foram reduzidas na era COVID-19. ^19^ Kiris et al. comparou a era pré-COVID à era COVID. Houve queda de 30,5% nas taxas de internação por IAMCSST ^19^ e outro estudo de Little et al. relatou uma redução de 21% na admissão por IAMCSST no Reino Unido. ^9^ Da mesma forma, foi comunicada uma redução de 40% em Espanha ^26^ e uma redução de 38% nos EUA nesses dados. ^27^ No presente estudo, não avaliamos esse parâmetro, mas o aumento do TIT e a diminuição das internações podem contribuir para o aumento das taxas de choque cardíaco, insuficiência cardíaca e mortalidade em pacientes positivos para COVID-19. O fato de o tempo porta-balão ser semelhante entre os dois grupos sugere que não houve atraso hospitalar.

Nosso centro é uma instituição de saúde experiente com alta circulação de pacientes. Amostras de swab nasal/faríngeo foram coletadas primeiro e a ICP primária foi aplicada a todos os pacientes em um período semelhante. Portanto, o presente estudo poderia prever dados da vida real sobre desfechos cardiovasculares adversos em pacientes com COVID-19.

### Limitações do estudo

Embora nosso estudo enfatize a associação entre o status positivo da COVID-19 e o IAMCSST, houve várias limitações. Este foi um trabalho retrospectivo e unicêntrico. Além disso, foram incluídos apenas pacientes submetidos a ICP primária. Embora o número total de pacientes fosse grande, a taxa no grupo positivo para COVID-19 foi inferior a 10% e permaneceu relativamente baixa. Além disso, a possibilidade de imprecisão nas amostras de swab pode ter afetado os resultados. Como a ultrassonografia intravascular não estava disponível em nosso hospital, o mau posicionamento do stent não pôde ser avaliado com clareza, o que pode ter levado à TS.

Este estudo não retira a importância de descrever as características evolutivas da população de COVID-19 com IAMCSST e merece estudos adicionais, inclusive já comparando o impacto da vacina nesses desfechos.

Nossos dados incluíram apenas os resultados intra-hospitalares. Não havia dados de acompanhamento disponíveis neste estudo. Serão necessários dados de longo prazo para determinar a associação entre a infecção por COVID-19 e desfechos cardíacos, como insuficiência cardíaca, trombose tardia do stent, hospitalização recorrente e morte.

## Conclusões

Em pacientes com IAMCSST, a coinfecção por COVID-19 apresenta piores desfechos cardíacos, atraso no tratamento e aumento das taxas de mortalidade. A ICP primária pode ser uma opção de tratamento eficaz e preferível para esses pacientes devido ao tempo porta-balão de acordo com as recomendações das diretrizes e que foi semelhante nos dois grupos. Além disso, os pacientes positivos para COVID-19 podem necessitar de terapia antitrombótica e anticoagulante mais agressiva devido ao aumento da atividade trombótica. Estudos adicionais são necessários para determinar o tratamento adequado e rápido de pacientes com COVID-19 e com IAMCSST e para identificar a causa subjacente de desfechos piores.
