# Supplementary material for: Desfechos Hospitalares do Infarto do Miocárdio com Supradesnivelamento do Segmento ST em Pacientes Positivos para COVID-19 que Passaram por Intervenção Percutânea Primária
Source: Arq Bras Cardiol. 2024 Jan 24;121(1):e20230258. [Article in Portuguese] doi: 10.36660/abc.20230258 (PMC11098568; doi:10.36660/abc.20230258)
Supplement: Translation

# Translation: In-Hospital Outcomes of ST-Segment Elevation Myocardial Infarction in COVID-19 Positive Patients Undergoing Primary Percutaneous Intervention

**Authors:** Nart Zafer Baytuğan, Hasan Çağlayan Kandemir, Tahir Bezgin

**Affiliations:** 1 Turkey Gebze Fatih State Hospital – Cardiology, Gebze – Turkey; 2 Turkey Kocaeli Devlet Hastanesi – Cardiology, Kocaeli – Turkey

**Keywords:** COVID-19, Myocardial Infarction, Mortality, Cardiogenic Shock

## Abstract

**Background:**

Concomitant coronavirus 2019 (COVID-19) infection and ST-segment elevation myocardial infarction (STEMI) are associated with increased adverse in-hospital outcomes.

**Objectives:**

This study aimded to evaluate the angiographic, procedural, laboratory, and prognostic differences in COVID-19-positive and negative patients with STEMI undergoing primary percutaneous coronary intervention (PCI).

**Methods:**

A single-center, retrospective, observational study was conducted between November 2020 and August 2022 in a tertiary-level hospital. According to their status, patients were divided into two groups (COVID-19 positive and negative). All patients were admitted due to confirmed STEMI and treated with primary PCI. In-hospital and angiographic outcomes were compared between the two groups. Two-sided p-values < 0.05 were accepted as statistically significant.

**Results:**

Of the 494 STEMI patients enrolled in this study, 42 were identified as having a positive dagnosis for COVID-19 (8.5%), while 452 were negative. The patients who tested positive for COVID-19 had a longer total ischemic time than did those who tested negative for COVID-19 (p=0.006). Moreover, these patients presented an increase in stent thrombosis (7.1% vs. 1.7%, p=0.002), length of hospitalization (4 days vs. 3 days, p= 0.018), cardiogenic shock (14.2% vs. 5.5 %, p= 0.023), and in-hospital total and cardiac mortality (p<0.001 and p=0.032, respectively).

**Conclusions:**

Patients with STEMI with concomitant COVID-19 infections were associated with increased major adverse cardiac events. Further studies are needed to understand the exact mechanisms of adverse outcomes in these patients.

## Introduction

Coronavirus disease 2019 (COVID-19), caused by coronavirus 2 (SARS-CoV-2), has been a pandemic since March 2020 and became a global health crisis in a short period of time worldwide. ^1 , 2^ Although COVID-19 primarily affects the respiratory system, it can lead to multiple organ involvement, systemic infection, vascular endothelial dysfunction, myocardial infarction, and death. ^3^ Systemic viral infections and hypoxia may trigger platelet activation, plaque rupture, and acute coronary syndromes with disruption of the vascular endothelial system. ^4 , 5^

COVID-19 affects both the arterial and venous systems, and leads to an increase in thrombotic activity. Thrombosis is a serious complication that often appears as a pulmonary embolism, cerebral infarction, and venous thromboembolism. Acute coronary syndrome, mesenteric and cerebrovascular ischemia, and renal artery thrombosis are less common. ST-segment elevation myocardial infarction (STEMI) usually occurs as a result of complete thrombotic occlusion of the coronary artery and requires a rapid diagnosis and reperfusion strategy. There is insufficient data on the effect of COVID-19 co-infection on the clinical outcomes of patients with STEMI.

We planned a retrospective, single-center study to evaluate the clinical, angiographic, laboratory, and procedural variables in COVID-19 positive patients with STEMI, as compared to COVID-19 negative and STEMI patients.

## Methods

### Patients population

An observational, single-center, retrospective study was conducted between November 2020 and August 2022. A total of 494 consecutive patients with STEMI, who were admitted to our catheterization laboratory, were included in the study. STEMI was defined based on the ST-segment elevation in two or more contiguous leads ≥ 0.2 mV or a new left bundle-branch block associated with a new onset of chest pain. ^6^ Angiographic diagnosis of occlusive coronary disease was performed in all patients, and none was treated with fibrinolysis. Patient data were obtained from hospital database records. Laboratory tests (complete blood count, inflammatory parameters, and biochemical parameters) were performed for all patients upon admission. Patients with non-STEMI, insufficient data of patients, and cardiac arrest were excluded from the study. In addition, patients who had not undergone percutaneous coronary intervention (PCI) (non-obstructive coronary artery lesions, vasospasms, or directed emergency bypass surgery) were not evaluated. This study was conducted according to the principles of the Declaration of Helsinki, and the local ethics committee approved the study protocol. The central illustration illustrates the study’s protocol.

### Clinical data collection

Nasal swab samples were collected from all patients in the emergency room or catheter laboratory before PCI. COVID-19 infection was confirmed by real-time reverse transcriptase-polymerase chain reaction (RT-PCR) assays and/or by evaluating symptoms with thoracic computed tomography (CT) imaging. They were categorized as COVID-19 negative and positive. Additional treatments (antibiotic, antiviral, etc.) were initiated using the current approaches in COVID-19 positive patients.

Left ventricular ejection function (LVEF) was measured using a 2D image from end-diastolic and end–systolic volume, using the modified Simpson’s method. Valve disorders were evaluated as moderate or severe regurgitation or stenosis of the mitral or aortic valves.

The clinical condition of the patients, additional disease history, smoking, length of hospital stay, stent thrombosis (ST), bleeding, cardiogenic shock, and mortality rate were recorded. Furthermore, the use of glycoprotein IIb-IIIa inhibitors, aspiration catheters, and intra-aortic balloon pumps were retrospectively analyzed. The time of symptom onset and door-to-balloon time were recorded for all patients. Coronary angiographic images were analyzed as responsible lesions and lesion type, post-PCI no-reflow, slow flow phenomenon, and residual lesion by two different expert cardiologists blinded to the patient’s data.

### Angiographic procedures

The procedures were performed according to the current guidelines, and the choice of radial or femoral artery, stent placement strategy, predilatation, postdilatation, use of glycoprotein IIb-IIIa inhibitors, and aspiration catheter were left to the operator’s discretion. Dual anti-aggregate therapy was initiated in all of the patients before the procedure. Acute and subacute ST were defined within 24 hours and 1 month after stent implantation, respectively, according to the Academic Research Consortium definitions. ^7^ All patients were administrated unfractionated heparin at a loading dose of 70-100 u/kg with an activated clotting time > 250 s.

### Definition of clinical outcomes

Patients were divided into two groups as COVID-19 positive and negative and compared according to length of hospital stay, major adverse cardiac events (MACE), major and minor bleeding, cardiogenic shock and in-hospital mortality rates. MACE was defined as myocardial infarction, stroke, heart failure, and/or death from cardiovascular disease **.** The bleeding designation was performed using the bleeding academic research consortium (BARC) definitions. ^8^ Cardiogenic shock was confirmed as a sign of poor end-organ perfusion in addition to systolic blood pressure below 90 mmHg for at least 30 minutes due to cardiac dysfunction.

### Statistical analysis

Categorical variables were expressed as numbers and percentages and compared using the chi-square and Fisher’s exact tests. The Kolmogorov–Smirnov test was used to determine whether the data had a normal distribution. Continuous variables with normal distribution were expressed as mean ± standard deviation and non-normal distribution as median and interquartile range. Unpaired Student’s t-test and Mann-Whitney U test were used to compare continuous variables with normal and without normal distributions, respectively. The parameters were analyzed using univariate and multivariate logistic regression analyses. The enter method was used in univariate analysis, and parameters with p-values < 0.1 were included in the multivariate logistic regression model. The multivariate model was adjusted for age, high sensitivity cardiac troponin I (hs-cTnI), ferritin, hemoglobin, D-dimer, COVID-19 (+), total ischemic time (TIT), and door-to-balloon time. Multivariate logistic regression models with clinically relevant variables was performed to detect independent predictors of MACE. The backward methods used multivariate logistic regression analysis, and a p-value < 0.05 was considered statistically significant. To analyze the correlation between D-dimer levels and post–PCI TIMI flow in COVID-19 positive STEMI patients, the Spearman correlation coefficient was calculated. All tests had a two-sided p-value < 0.05 and were accepted as statistically significant. Data were analyzed using the SPSS 22.0 version (SPSS Inc., Chicago, Illinois, USA).

## Results

The present study included 494 consecutive patients, 298 men (60.4%) and 196 women (39.6%), with a median age of 59 (42-80). Baseline demographic, clinical, and laboratory parameters of the study population are shown in Table 1 . COVID-19 positive group was older and had a similar prevalence of smoking, hypertension, coronary artery disease, congestive heart failure, PCI history, and atrial fibrillation. Diabetes mellitus (DM) and chronic obstructive pulmonary disease (COPD) were more common in the COVID-19 positive group ( Table 1 ).

**Table 1:** – The baseline demographic, clinical and laboratory characteristics of the patients

	COVID-19 negative (n=452, 91.5%)	COVID-19 positive (n=42, 8.5%)	p-value
**Age**	58 (41-82)	72 (58-83)	<0.001
**Gender (Female), n (%)**	178 (39.3)	18 (42)	0.214
**Smoking, n (%)**	189 (41.8)	15 (35.7)	0.515
**Symptoms**			
Pain	383 (84.7)	28 (66.6)	
Dyspnea	52 (11.5)	11 (26.1)	
Cardiac arrest	8 (1.7)	2 (4.7)	
Other	9 (2)	1 (2.3)	
**Past medical history**			
DM, n (%)	60 (13.2)	18 (42.8)	0.017
HT, n (%)	130 (28.7)	15 (35.7)	0.344
Previous CHF, n (%)	18 (3.9)	3 (7.1)	0.245
Previous CAD, n (%)	38 (8.4)	3 (7.1)	0.431
Previous PCI, n (%)	51 (11.2)	6 (14.2)	0.795
AF, n (%)	24 (5.3)	2 (4.7)	0.712
COPD, n (%)	68 (15.0)	18 (42.8)	0.021
**STEMI presentation**			
Anterior/LBBB, n (%)	219 (48.4)	23 (54.7)	
İnferior, n (%)	168 (37.1)	16 (38.0)	
Lateral, n (%)	34 (7.5)	3 (7.1)	
Posterior, n (%)	31 (6.8)	0 (0)	
**Echocardiographic features**			
LVEF (%)	45.7 ± 7.1	40.9 ± 8.2	0.009
Valve disease, n (%)	49 (10.8)	5 (11.9)	0.341
**Killip classification**			
Killip I, n (%)	304 (67.2)	18 (42.8)	0.058
Killip II, n (%)	92 (20.3)	10 (23.8)	0.267
Killip III, n (%)	31 (6.8)	7 (16.6)	0.034
Killip IV, n (%)	25 (5.5)	6 (14.2)	0.023
**Laboratory values**			
hs-cTnI, ng/mL	1126 (215-32100)	12742 (453-48756)	<0.001
Creatinine, mg/dL	0.8 (0.5-2.2)	0.9 (0.6-2.4)	0.779
Glucose, mg/dL	105 (79-207)	144 (106-321)	<0.001
AST mg/dL	23 (18-30)	32 (22-47.5)	<0.001
ALT mg/dL	26 (16-29)	28 (18-30)	0.208
D-dimer ng/mL	0.52 (0.3-1.1)	2.45 (0.8-7.5)	<0.001
Ferritin, ng/mL	174 (101-316)	421 (134-879)	<0.001
hs-CRP, mg/L	9.6 (2.3-45.3)	48.9 (25.1-155.9)	<0.001
WBC x10 ^3^ /µL	6810 ± 2617	14820 ± 4321	<0.001
Hemoglobin, g/dL	13.2 ± 1.6	13.4 ± 2.6	0.447
Thrombocyte x10 ^3^ /µL	235 ± 81	242 ± 129	0.344
Lymphocyte x10 ^3^ /µL	2.1 ± 0.9	0.9 ± 0.7	<0.001

*AF: atrial fibrillation; ALT: alanine aminotransferase; AST: aspartate transaminase; CAD: coronary artery disease; CHF: chronic heart failure; COPD: chronic obstructive pulmonary disease; DM: diabetes mellitus; LBBB: left bundle branch block; LVEF: left ventricular ejection fraction; hs-CRP: high-sensitivity C-reactive protein; hs-cTnI: high-sensitivity cardiac troponin I; HT: hypertension; PCI: percutaneous coronary intervention; WBC: white blood cell.*

### Laboratory findings

According to laboratory assays on admission, COVID-19 positive patients had higher levels of inflammatory markers (hs-cTnI, D-dimer, hs-CRP, ferritin, white blood cell count), fasting blood glucose, and AST levels. ALT, hemoglobin, creatinine, and thrombocyte counts were similar in both groups ( Table 1 ).

### Coronary angiographic findings

The average stent diameter and total stent length were similar in both groups, and the shortest and longest stents sizes were 8 and 56 mm, respectively ( Table 2 ). The rate of multivessel PCI, bifurcation lesion, pre-dilatation, post-dilatation, overlap stent, and residual lesion ratio were found to be parallel in the groups. In addition, baseline TIMI 0-1 and post-PCI TIMI 3 flow ratios were similar ( Table 2 ). The COVID-19 positive group showed a greater use of glycoprotein IIb/IIIa inhibitor and aspiration device. The no-reflow phenomenon was higher in COVID-19 positive patients, and post-PCI TIMI 3 flow and D-dimer levels were negatively correlated ( Figure 1 ). There was no difference between the groups in terms of intra-aortic balloon pump use.

**Figure 1:** – Correlation between D-Dimer levels and post-PCI TIMI flow in COVID-19 positive STEMI patients.

### In-hospital outcomes

Patients with COVID-19 and STEMI had higher in-hospital cardiac and overall mortality, ST, and cardiogenic shock ( Table 3 , Figure 2 ). In multivariate analysis, older age, COVID-19 infection, D-dimer, ferritin, hemoglobin, and hs-cTnI levels were an independent predictor of MACE ( Table 4 ).

**Table 3:** – In-hospital outcomes of patients

	COVID-19 negative (n= 452)	COVID-19 positive (n= 42)	p-value
**Cardiogenic shock, n (%)**	25 (5,5)	6 (14,2)	0,023
**Total hospitalization (day)**	3 (2-6)	4 (3-11)	0,018
**BARC grades 0-1, n (%)**	14 (3,0)	2 (4,7)	0,372
**BARC grades 2-4, n (%)**	2 (0,4)	0 (0)	0,571
**Stent thrombosis, n (%)**	8 (1,7)	3 (7,1)	0,002
**In-hospital mortality, n (%)**	29 (6,4)	10 (23,8)	<0,001
**Causes of mortality**			
Cardiac, n (%)	25 (5,5)	5 (11,9)	0,032
Sepsis, n (%)	-	2 (4,7)	
Multi-organ dysfunction, n (%)	3 (0,6)	1 (2,3)	
Acute Respiratory failure, n (%)	-	1 (2,3)	
Other, n (%)	1 (0,2)	1 (2,3)	

**Figure 2:** – In-hospital outcomes with study cohort..

**Table 4:** – Univariate and multivariate analysis to predict MACE

Variables	Univariate		p value	Multivariate		p value
	HR	[95%CI]		HR	[95%CI]	
**Age**	1.041	1.031-1.052	<0.001	1.023	1.010-1.032	0.003
**Male gander**	0.610	0.382-0.871	0.041			
**Hypertension**	2.421	1.041-3.080	0.026			
**Diabetes mellitus**	0.901	0.871-1.012	0.070			
**CHF**	1.376	0.954-2.001	0.002			
**hs-cTnI**	2.581	1.557-4.280	<0.001	2.466	1.422- 4.263	<0.001
**Creatinine**	0.452	0.181-1.103	0.778			
**hs-CRP**	1.532	1.062-2.216	<0.001			
**Ferritin**	1.371	0.952-2.009	<0.001	1.221	0.816-1.837	0.009
**D-dimer**	0.169	0.028-1.222	<0.001	0.244	0.033-1.952	<0.001
**Hemoglobin**	0.900	0.814-1.011	0.007	1.012	0.833-1.205	0.002
**COVID-19 (+)**	3.921	2.051-7.472	<0.001	3.431	1.732-6.825	<0.001
**LVEF**	2.106	1.433-3.092	0.032			
**Multi-vessel disease**	1.621	0.982-2.688	0.331			
**IABP use**	1.004	1.001-1.102	0.002			
**Gp IIb-IIIa inhibitor use**	1.786	1.055-3.012	0.003			
**TIT**	1.344	0.957-1.880	<0.001	1.228	0.811-1.832	<0.001
**Door-to-balloon time**	2.588	1.553-4.287	<0.001	2.466	1.422- 4.260	<0.001

*CHF: chronic heart failure; Gp: glycoprotein; IABP: intra-aortic balloon pump; LVEF: left ventricular ejection fraction; TIT: total ischemic time; hs-CRP: high-sensitivity C-reactive protein; hs-cTnI: high-sensitivity cardiac troponin I.*

COVID-19 positive patients had longer in-hospital stay, and these patients had a high rate of Killip class III and IV upon hospital admission. Echocardiographic evaluation showed that COVID-19 negative patients had a higher LVEF. There was no difference between the groups due to valve disease ( Table 1 ).

Door-to-balloon times were within 48 min and were similar in both groups. However, the TIT was significantly higher in COVID-19 positive patients ( Table 2 , Figure 3 ). Both door-to-balloon time and TIT were independent predictors of in-hospital MACE ( Table 4 ). There were no significant differences between the groups in the BARC grades for bleeding ( Table 3 ).

**Figure 3:** – Total ischemic time and door-to-balloon time in COVID-19 positive and negative groups.

## Discussion

An observational study was planned based on the experience of a single center with high patient density. These results demonstrated that COVID-19 positive, STEMI patients showed a significantly increased rate of in-hospital cardiac and total mortality, cardiogenic shock, and hospitalization stay. Similar to the present study, current literature findings suggest that patients with COVID-19, diagnosed with STEMI, had higher rates of in-hospital mortality and cardiogenic shock. ^9 , 10^

The high MACE rate in these patients may be due to many reasons. The high prevalence of DM and COPD may have contributed to poor outcomes in this group. In addition, increased systemic infections due to COVID-19 itself have led to increased mortality and MACE.

Despite similar door-to-balloon times, there was a significant difference in hs-cTnI levels between the groups. Possible reasons for elevated hs-cTnI in COVID-19 positive patients include longer TIT and consequent delay in correct diagnosis. Delayed PCI and increased ischemic time may contribute to elevated cardiac enzymes. Cardiac complications may account for approximately 40% of all deaths in COVID-19 patients. ^11^ Increased inflammation, hypercoagulation, progressive respiratory failure, hypoxia, myocarditis, and direct toxic effects of the virus on host cells can lead to cardiac damage. In addition, post PCI no reflow phenomenon was significantly more common in the COVID-19 group, which may affect post-procedural perfusion and lead to a cardiac enzyme increase. Therefore, heart failure and cardiogenic shock may be more common in these patients, which may explain the high MACE, Killip class, and hs-cTnI levels. ^12^ We consider that non-cardiac causes in COVID-19 patients, such as sepsis, pulmonary embolism, and multiorgan failure, may contribute to the increase in hs-cTnI levels.

Hypoxemia, vasoconstriction, and impaired oxygenation are common findings in COVID-19 disease, thus hospital stay is prolonged, presenting an advanced risk of the patient’s multi-organ failure, bacterial infections, sepsis, and thrombosis. ^13^ Systemic inflammation activates the pro-thrombotic cascade and disrupts endothelial function, thus increasing the risk of thrombosis and related complications. ^14^ Elevated inflammatory markers are associated with increased mortality rates. ^14^ Increased inflammatory response and hemodynamic changes have proven to increase the risk of plaque rupture and related myocardial infarction in influenza virus infection. ^15^ We found that hs-CRP, ferritin, and D-dimer levels were independent predictors of MACE development. In addition, the inflammatory parameters were significantly higher in the COVID-19-positive group, which is similar to these results.

In our study, markers indicating increased thrombotic activity, such as multi-vessel thrombus, ST, no-reflow phenomenon, the use of GP IIb/IIIa inhibitors, and the aspiration device, were detected more frequently in COVID-19 positive patients. A higher thrombus burden in COVID-19 patients is associated with an increased risk of adverse cardiac events and death. ^16^ In addition, distal embolization of the thrombus may disrupt the microvascular flow, leading to a no reflow-slow flow phenomenon and an increase in the area of infarction. ^17^

In a study by Choudry et al., inflammatory parameters were higher in patients with STEMI and COVID-19, and D-dimer levels were correlated with thrombus grade. ^18^ A similar result was found in another study, with a positive correlation between the thrombus grade and D-dimer levels in STEMI patients. ^19^ Our study found a parallel finding with D-dimer levels and a negative correlation with the post-PCI coronary flow. Additionally, the mortality benefit of anticoagulant use in a large group of COVID-19 patients without myocardial infarction was also demonstrated. ^20^

TIT is an important criterion affecting mortality in STEMI patients. ^21^ Prolongation of this period reduces myocardial salvage and increases the area of infarction and subsequent long-term mortality. ^21^ Current guidelines recommend primary PCI with a door-to-balloon time of 90 minutes if the patient presents to a PCI capable hospital. ^22 , 23^ Onder et al. showed that the mean time from the onset of symptoms of STEMI to the first medical contact during the COVID-19 pandemic was 318 minutes, ^24^ and another study by Abdelaziz et al. demonstrated that this time was an average of 227 minutes. ^25^ Our study found that this time was 390 minutes in COVID-19 positive patients, and it was significantly longer when compared to COVID-19 negative patients. The prolongation of the first medical contact may have affected the increased MACE observed in the COVID-19 positive group ( Table 4 ).

STEMI admissions to medical centers were diminished during the COVID-19 era. ^19^ Kiris et al. compared the pre-COVID era with the COVID era itself. There was a 30.5% drop in STEMI hospitalization rates, ^19^ and another study by Little et al. reported a 21% reduction in STEMI admission in the United Kingdom. ^9^ Similarly, a 40% reduction was reported in Spain ^26^ and a 38% reduction in the US in these data. ^27^ The present study did not evaluate this parameter, but increased TIT, and decreased admissions may contribute to increased cardiac shock, heart failure, and mortality rates in COVID-19 positive patients. The fact that the door-to-balloon time was similar between the two groups suggests that there was no in-hospital delay.

Our center is an experienced healthcare institution with a high patient circulation. Nasal swab/pharyngeal samples were collected first, and primary PCI was applied to all patients in a similar time frame. Therefore, the present study could predict real-life data on adverse cardiovascular outcomes in COVID-19 patients.

### Limitations of the study

Although our study emphasized the association between COVID-19 positive status and STEMI, there were several limitations. This was a retrospective and single-center work. In addition, only patients undergoing primary PCI were included. Although the total number of patients was large, the rate in the COVID-19 positive group was less than 10% and remained relatively low. In addition, the possibility of inaccuracy in swab samples may have affected the results. Since intravascular ultrasonography was not available in our hospital, stent malposition could not be evaluated clearly, which may have led to ST.

This study does not remove the importance of describing the evolutionary characteristics of the COVID-19 population with STEMI and deserves further study, including already comparing the impact of the vaccine on these outcomes

Our data included only the in-hospital results. No follow-up data were available in this study. Long-term data will be required to determine the association between COVID-19 infection and cardiac outcomes, such as heart failure, late stent thrombosis, recurrent hospitalization, and death.

## Conclusions

In patients with STEMI, COVID-19 co-infection presents with poorer cardiac outcomes, delayed treatment, and increased mortality rates. Primary PCI may be an effective and preferable treatment option for these patients because of the door-balloon time according to the recommendations in the guidelines and which was similar in the both groups. In addition, COVID-19 positive patients may require more aggressive antithrombotic and anticoagulant therapy because of their increased thrombotic activity. Additional studies are needed to determine the appropriate and rapid treatment of COVID-19 patients with STEMI and to identify the underlying cause of poorer outcomes.

**Figure:** Central Illustration: In-Hospital Outcomes of ST-Segment Elevation Myocardial Infarction in COVID-19 Positive Patients Undergoing Primary Percutaneous Intervention

**Table 2:** – Angiographic and procedural findings of the patients

	COVID-19 negative (n= 452)	COVID-19 positive (n= 42)	p-value
**Total coronary intervention, n (%)**	471	44	
**Target lesion, n (%)**			
LAD	216 (47.7)	23 (54.7)	
Cx	97 (21.4)	6 (14.2)	
RCA	123 (27.2)	11 (26.1)	
LMCA	3 (0.6)	0 (0)	
Graft PCI	11 (2.4)	0 (0)	
Multi-vessel PCI	19 (4.2)	2 (4.7)	0.680
Bifurcation lesion	46 (10.1)	3 (7.1)	0.492
**Lesion Type, n (%)**			
Type A	48 (10.6)	6 (14.2)	
Type B	216 (47.7)	24 (57.1)	
Type C	188 (41.5)	12 (28.5)	
**Total stent length (mm)**	26.1 ± 7.0	30.1 ± 6.7	0.319
**Anti-aggregate therapy**			
Clopidogrel, n (%)	108 (23.8)	19 (45.2)	
Tigacrelor, n (%)	221 (48.8)	18 (42.8)	
Prasuqrel, n (%)	123 (27.2)	5 (11.9)	
**Average stent diameter (mm)**	3.0 ± 0.3	3.0 ± 0.2	0.473
**Overlap stent, n (%)**	32 (6.1)	2 (9.5)	0.815
**Residual lesion, n (%)**	4 (0.8)	0 (0)	0.910
**Baseline TIMI 0-1 flow, n (%)**	401 (88.7)	40 (95.2)	0.141
**Post-PCI TIMI 3 flow, n (%)**	389 (86.0)	34 (80.9)	0.576
**Post- PCI no-reflow, n (%)**	34 (7.5)	8 (19)	0.043
**Gp IIb-IIIa inhibitor use, n (%)**	58 (12.8)	12 (28.5)	0.026
**Aspiration thrombectomy use, n (%)**	78 (17.2)	14 (33.3)	0.032
**IABP use, n (%)**	15 (3.3)	4 (9.5)	0.207
**Door-to-balloon time minutes [median IQR]**	46 (30-72)	48 (36-77)	0.240
**TIT, minutes [median IQR]**	270 (110-670)	390 (180-960)	0.006

*Cx: circumflex artery; Gp: glycoprotein; IABP: intra-arterial balloon pump; IQR: inter quarter range; LAD: left anterior descending artery; LMCA: left main coronary artery; PCI: percutaneous coronary intervention; RCA: right coronary artery; TIT: total ischemic time.*
